# Supplementary material for: MetaRibo-Seq measures translation in microbiomes
Source: Nat Commun. 2020 Jun 29;11:3268. doi: 10.1038/s41467-020-17081-z (PMC7324362; doi:10.1038/s41467-020-17081-z)
Supplement: Supplementary file 10 — Supplementary Data 7 [file 41467_2020_17081_MOESM10_ESM.zip › File2/Confidence_VeryHigh_Taxonomy/184389_out.krona.html]

Javascript must be enabled to view this page.

members
magnitude
magnitudeUnassigned
count
unassigned
taxon
rank

184389\_out

4

4
2
superkingdom

phylum
1
32066

1
203490
class

203491
1
order

family
1129771
1

1
32067
genus

species
1

SRS077308\_contig\_number\_11819
712359

1
976
phylum

1
200643
class

order
1
171549

1
171550
family

239759
1
genus

1
28117

SRS018888\_contig\_number\_611
species

2
1224
phylum

class
28216
2

206351
2
order

family
2
481

genus
482
2


SRS013949\_contig\_number\_contig-100\_962.142271SRS056090\_contig\_number\_7010
2
1739411
species
